# “Optical and Surface Enhanced Raman Scattering properties of Ag modified silicon double nanocone array”

**DOI:** 10.1038/s41598-017-12423-2

**Published:** 2017-09-21

**Authors:** L. Mehrvar, M. Sadeghipari, S. H. Tavassoli, S. Mohajerzadeh, M. Fathipour

**Affiliations:** 1 Laser and Plasma Research Institute, Shahid Beheshti University, G. C., Evin, Tehran, 19839, Iran, Tehran, 1483963113 Iran; 2 Thin Film and Nanoelectronics Lab, Nanoelectronics Center of Excellence, School of Electrical and Computer Engineering, University of Tehran, Tehran, 143957131 Iran; 3 MEMS & NEMS Lab, Department of Electrical and Computer Engineering, University of Tehran, Tehran, 143957131 Iran

## Abstract

Surface enhanced Raman scattering (SERS) systems with large number of active sites exhibit superior capability in detection of low concentration analytes. In this paper, we present theoretical as well as experimental studies on the optical properties of a unique hybrid nanostructure, Ag NPs decorated silicon double nanocones (Si-DNCs) array, which provide high density of hot spots. The Si-DNC array is fabricated by employing electron beam lithography together with plasma etching process. Multipole analysis of the scattering spectra, based on the multipole expansion theory, confirms that the toroidal dipole moment dominates over other electric and magnetic multipole moments in the Si-DNCs array. This response occurs as a result of generating current densities flowing in opposite directions and consequently generating H-field vortexes inside the nanocones. Moreover, SERS applicability of this type of nanostructure is examined. For this purpose, the Si-DNCs array is decorated with Ag nanoparticles (NPs) by means of electroless deposition method. Simulation results indicate that combination of multiple resonances, including LSPR resonance of Ag NPs, longitudinal standing wave resonance of Ag layer and inter-particle interaction in the gap region, result in a significant SERS enhancement. Our experimental results demonstrate that Si-DNC/Ag NPs array substrate provides excellent reproducibility and ultrahigh sensitivity.

## Introduction

Surface enhanced Raman spectroscopy (SERS) is a powerful analytical tool for detection and identification of chemical^1–4^ and biological^5–8^ materials. High local electromagnetic field (EM) enhancement caused by localized surface plasmon excitation, called a “hot spot”^9–11^, leads to the enhancement of the Raman scattering. Besides, higher EM field enhancements are needed for detection of lower analyte concentrations. Interestingly, extremely high EM enhancement can be created in the gap between metal nanoparticles (NPs) and in the vicinity of metallic tips^12,13^ (due to “lightning effect”).

Metallic NPs decorated on the side walls of the dielectric nanostructures is one of the high performance SERS substrates^14,15^. In such hybrid nanostructures, the strong near electric field enhancement originates from excitation of multiple resonances. As a consequence, the Raman signal of analyte molecules in the vicinity of hotspots is dramatically enhanced. Various methods have been developed for manufacturing of these dielectric nanostructure arrays. Combination of lithography methods with reactive ion etching (RIE) is an impressive technique used for manufacturing dielectric nanostructure arrays^16,17^.

These high-index dielectric nanostructure arrays have received great attention for their interesting optical properties to control light–matter interaction^18–20^. Actually, two families of multipoles including electric and magnetic multipoles can be excited in these dielectric nanostructures. The third kind of electromagnetic moments are toroidal multipoles which are characterized by vortex distributions of the magnetic moments. Toroidal multipoles have been drawing a lot of attentions because of their interesting electromagnetic properties and various applications such as polarization transformers^21^, circular dichroism (CD)^22^ and low-threshold lasing^23^. However, there are no reports on the excitation of toroidal moments in the dielectric nanostructures.

In this manuscript, we report a new SERS substrate, Ag NPs decorated Si-DNCs array, which provide ultra-high electromagnetic field enhancement. Actually, this unique SERS substrate creates multiple types of hot spot sources simultaneously, including (I) interaction mode between nanocone pairs, (II) LSPR modes of AgNPs, and (III) LSPR mode in the vicinity of metallic tips (lightning rod effect), which result in high concentration of active sites and consequently high amount of enhancement factor (EF). Furthermore, optical properties of the fabricated Si-DNCs array are investigated by experimental measurements and theoretical calculations and physical cause of its response is explained. The optical characterization of this new nanostructure shows that the toroidal dipole (TD) moment dominates over other electric and magnetic multipole moments which can produce many interesting properties such as nonreciporocal refraction^24^ and magnetoelectric effect^25^.

## Fabrication procedures

### Fabrication of silicon double nanocone array

**Figure 1:**
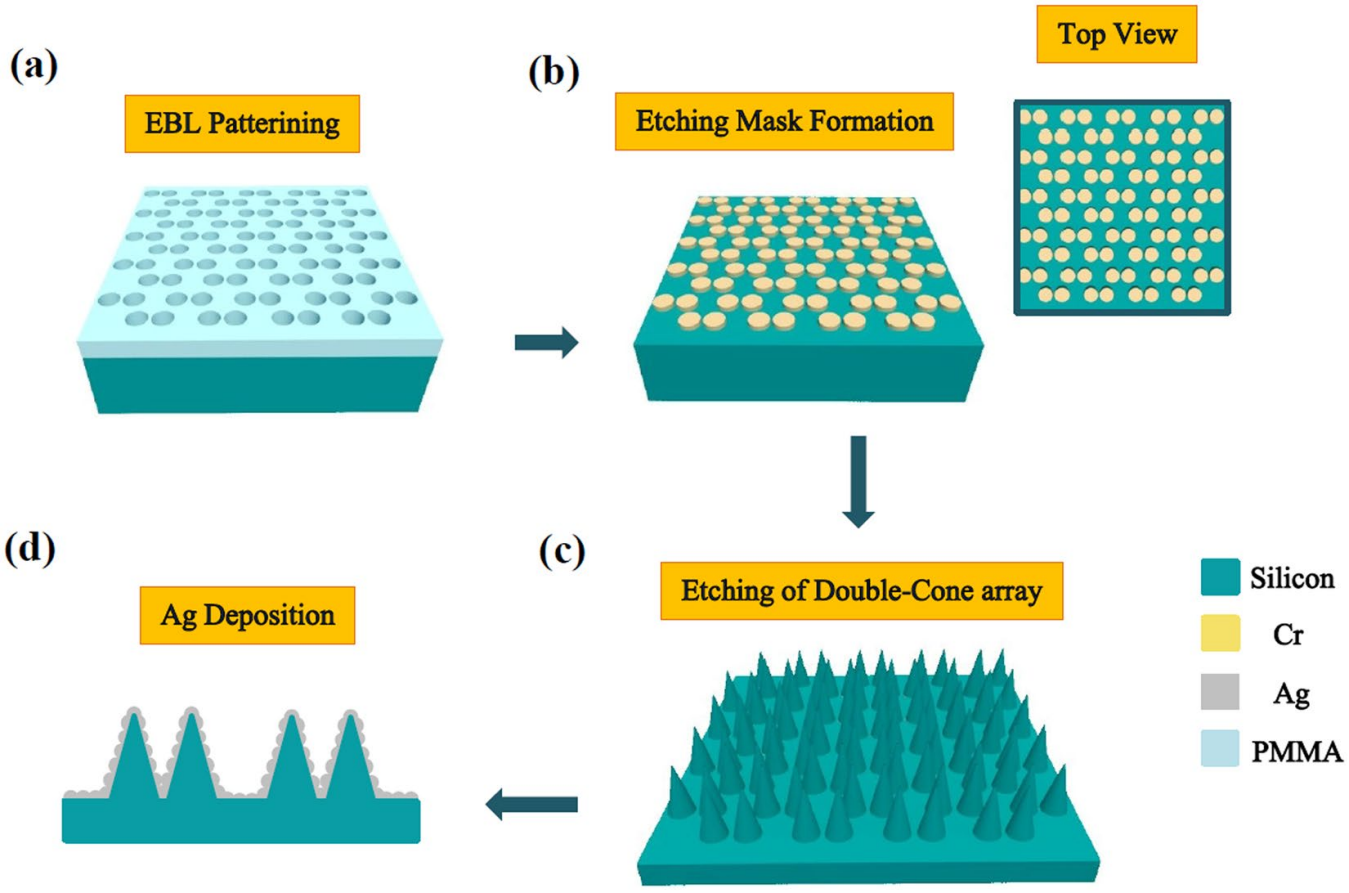
The schematic illustration of fabrication process for the SERS substrates. (**a**) generating double nanohole pattern using e-beam lithography (EBL) on PMMA (**b**) deposition of chromium (Cr) thin film on a substrate and lift off process, inset shows the top view of the created mask (**c**) the formation of DNC array using the RIE process. (**d**) Chemical deposition of Ag NPs on the DNC array.

**Figure 2:**
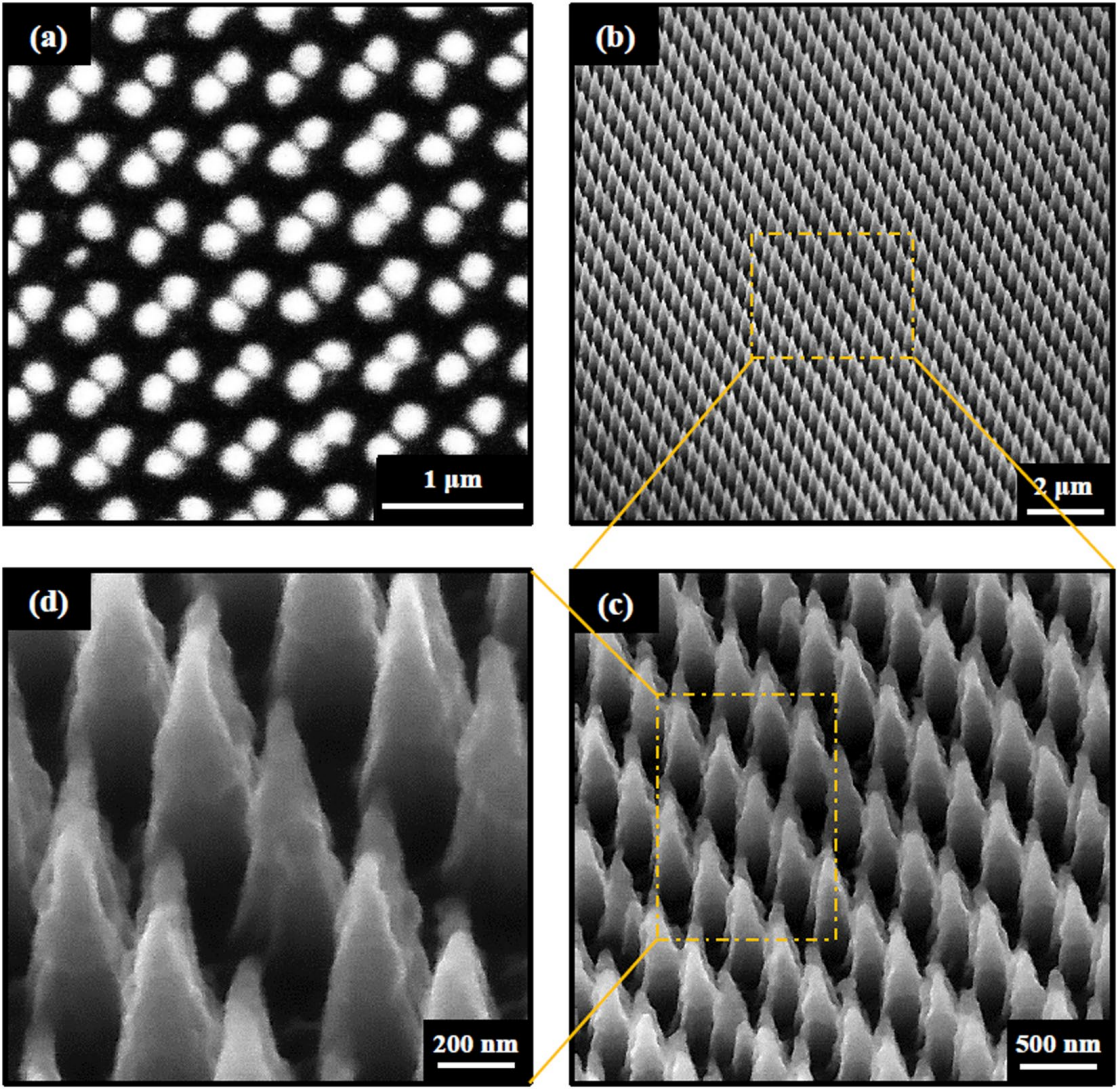
(**a**) The top view and (**b**) 70° tilt view scanning electron microscopy (SEM) images of DNC array. (**c**) Magnified image of (**b**). (**d**) Magnified image of (**c**).

An array of silicon DNCs has been realized by a multistep process on p-type silicon (100) substrates. Fabrication of this array is performed using three main steps: (i) the EBL of a deposited resist on a silicon substrate, (ii) deposition of Cr layer and (iii) chemical plasma etching. All fabrication steps of DNC array is schematically demonstrated in Fig. 1 (all panels). First, silicon substrate is spin coated with 300 nm thick layer of Poly Methyl Methacrylate (*PMMA*) as an EBL-resist. Then, double hole array structure is patterned on the PMMA resist using EBL method, Fig. 1a shows this structure after double hole patterning. In the next step, the PMMA is developed in a solution of 1:3, methyl-isobutyl-ketone (MIBK) and Isopropyl Alcohol (IPA) mixture, for 1min. Then, a 20 nm thick layer of Cr is deposited on the substrate, by means of an electron beam evaporator at a base pressure of 10^−6^ Torr to act as hard mask in the deep reactive ion etching (DRIE) process. By DRIE system, three-dimensional DNC array are fabricated in an RF plasma (13.56 MHz) environment equipped with O_2_, SF_6_, and H_2_ gases with sequential etching/passivation subcycles. Desired shaped will be achieved by adjusting the gas mixture, power, and duration of the etching/passivation subcycles. During the passivation step, a mixture of O_2_/H_2_ with a trace of SF_6_ value is introduced into the reactor while duration of this subcycle was 50 seconds (s) with a plasma power of 250 W. Etching was achieved using only SF6 as the fed gas with a flow of 150 SCCM with a short duration of 9 s and the plasma power of 110 W. Desired array was fabricated with repetition of 15 cycles of these two subcycles as shown in Fig. 2. This etching process allows an accurate control on structures height and thickness for tuning optical properties of devices based on silicon structures. The images are taken using a TSCAN MIRAII FESEM. A plane top view SEM of the fabricated DNC array is shown in Fig. 2a.

### Chemical deposition of silicon double nanocone array

**Figure 3:**
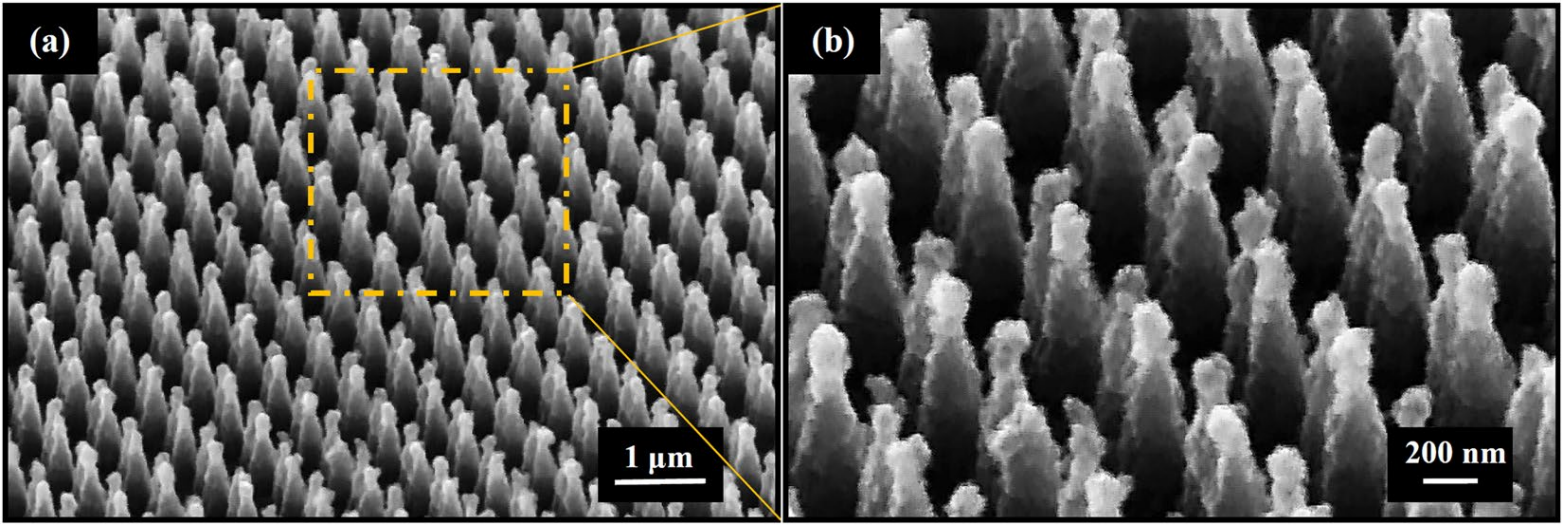
(**a**) 45° tilt view SEM images captured from DNC decorated with AgNPs with duration time of 20. (**b**) Magnified image of (**a**).

The manufactured Si DNCs substrates are decorated with silver nanoparticles (AgNPs) using electroless deposition method^26,27^. The DNC substrates are submerged into an aqueous AgNO3/HF (0.5 mM AgNO3, 9% HF) solution for 20s time intervals at room temperature. The DNC Samples immediately are rinsed with deionized water and then dried under a gentle stream of nitrogen. The SEM images of DNCs/AgNPs heterostructure are shown in Fig. 3. Images show that the AgNPs with larger size are deposited at the tip of the cones and smaller ones are deposited at the cone walls. Actually, raising the time duration increases the diameter of the AgNPs through the whole nanocone length as well as AgNPs aggregation on the tip of nanocones. In our previous work, it was demonstrated that the deposition time of 20 s provides uniform distribution of Ag NPs and more total number of hot spots in comparison to the substrates with higher deposition time^28^. For this reason, the Si DNCs array is deposited under 20s time deposition in this manuscript.

## Results and Discussion

### Optical response characteristics of DNCs array

**Figure 4:**
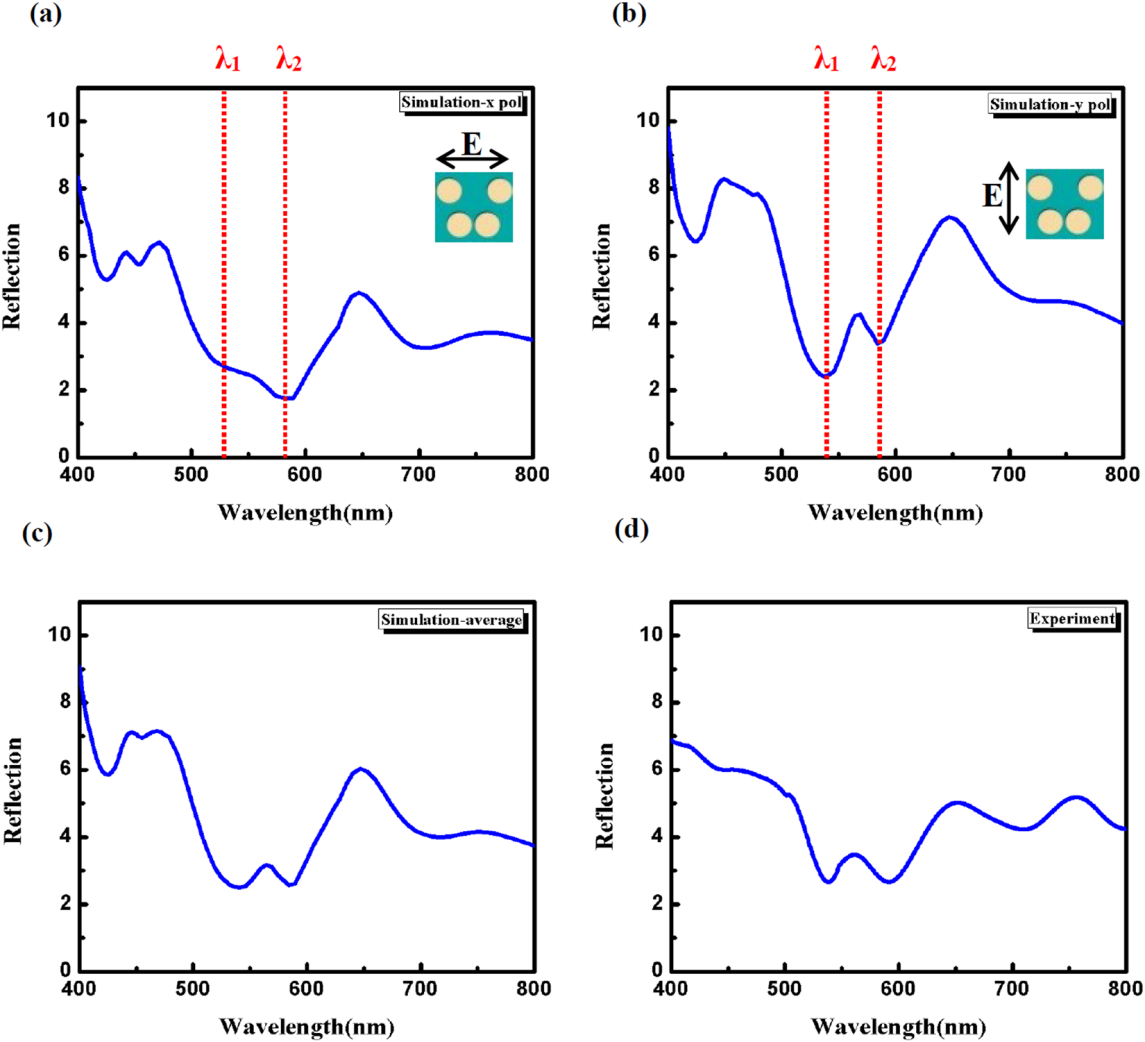
The simulated reflection spectrum of DNC array for incident (**a**) X-polarized and (**b**) Y-polarized light. Inset show the top view of unit cell in the simulations. (**c**) The simulated reflection spectrum of DNC array for unpolarized light achieved by averaging the X and Y polarized reflection spectra. (**d**) The experimental reflection spectrum under unpolarized light. It is seen that the calculated spectrum is similar to the experimental result.

To characterize the optical properties of the fabricated DNC arrays, the experimental reflection spectrum is measured and shown in Fig. 4d. As shown, the optical reflection response of this dielectric nanostructure is a complex combination of multiple resonances. Therefore, the optical response of this nanostructure is simulated to enable understanding the nature of each resonance. The reflection spectra of DNC array for the incident X-polarized, Y-polarized and unpolarized light are simulated and shown in Fig. 4a,b and c, respectively. The reflection spectrum for unpolarized light is achieved by averaging the X- and Y-polarized reflection spectra. As seen, a good agreement is obtained between theoretical and experimental results by comparing Fig. 4 part c with part d. Multiple resonances exist in the spectra, however two dips at 528 and 578 nm for X-polarized light and at 536 and 584 nm for Y-polarized light are demonstrated in Fig. 4a and b.

**Figure 5:**
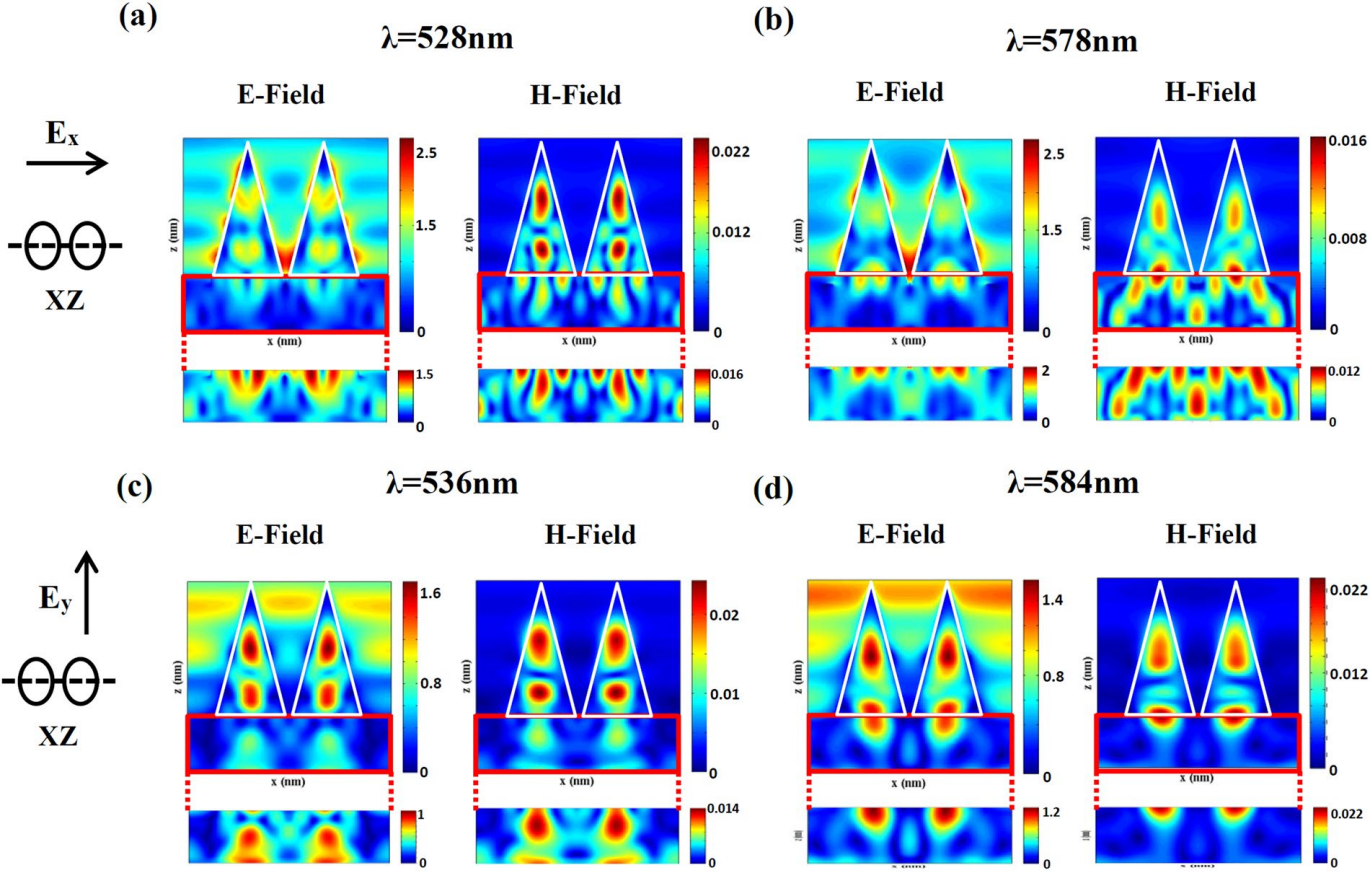
The cross section E- and H- field maps for incident X-polarized light at the resonances (**a**) λ_1_ = 528nm and (**b**) λ = 578nm (specified in Fig. 4a). The cross section E- and H- field maps for incident Y-polarized light at the resonances (**c**) λ_1_ = 536nm and (**d**) λ = 584nm (specified in Fig. 4b). It is seen that both electric and magnetic field resonances are simultaneously excited. Below is enlargement of the Si layer.

To characterize the nature of these resonant modes in the reflection spectra, near electric and magnetic fields are computed (at the two specified dips of each polarizations reflection spectrum as shown in Fig. 4, λ_1_ = 528nm and λ_2_ = 578nm for incident X-polarized light, λ_1_ = 536nm and λ_2_ = 584nm for incident Y-polarized light). It is seen that both electric and magnetic field resonances are spectrally overlapped. The electric and magnetic field enhancement are clearly observed from the near field distributions (shown in Fig. 5). The electric field enhancement is observed in three regions including (i) on the sidewall, (ii) inside the NCs and (iii) between two NCs. However, magnetic field enhancement is mostly confined inside the silicon NCs. These near electric and magnetic field enhancement is caused by the excitation of Mie resonances which provide potential applications for field enhanced surface spectroscopy^29^.

**Figure 6:**
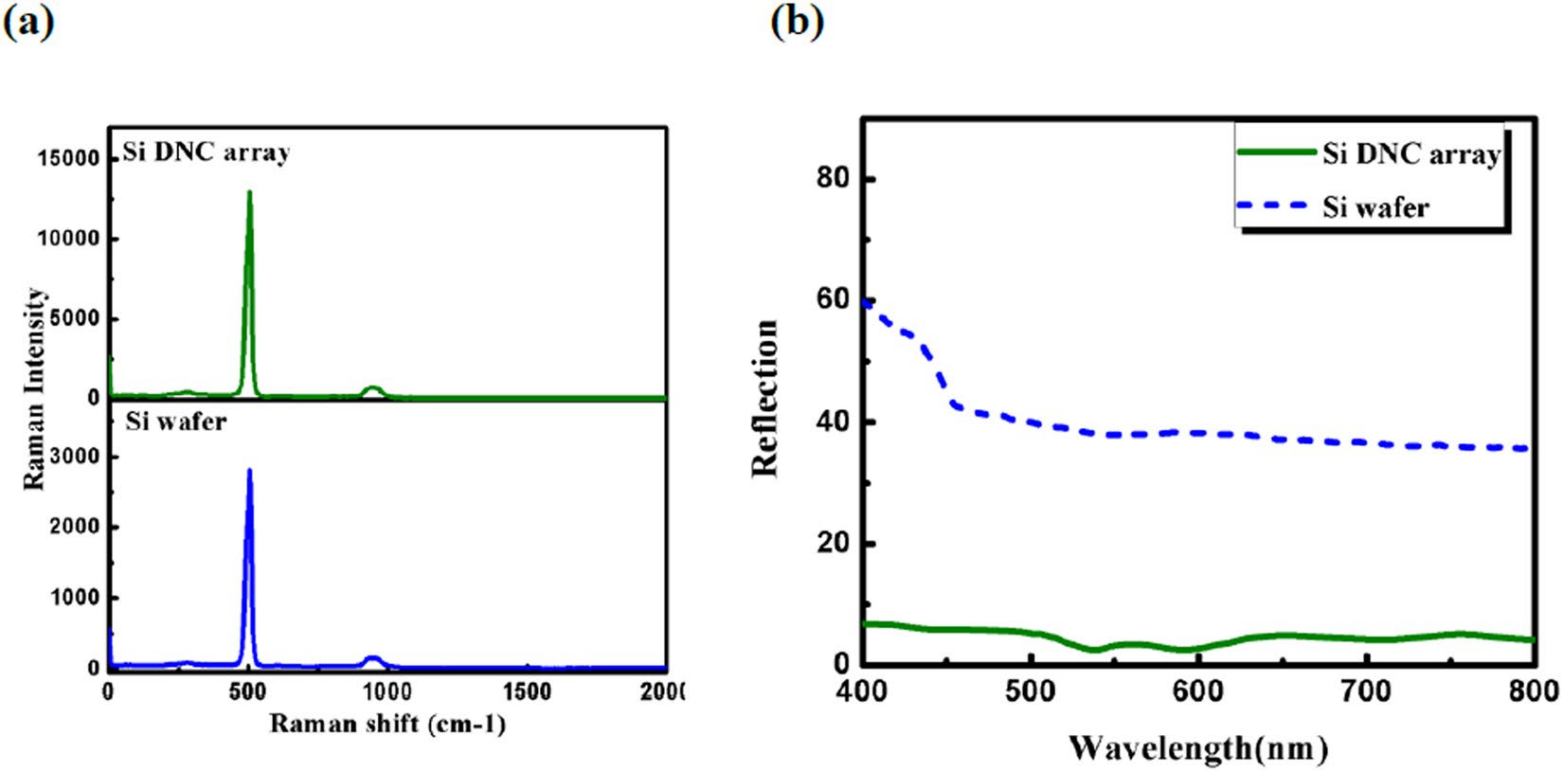
(**a**) Raman spectra of bare DNC array and silicon wafer. (**b**) Comparison between reflectance spactra of planar Si wafer and DNC array.

As mentioned, the near electric field enhancement leads to amplification of the Raman signal. So to estimate the average local electric field enhancement, the Raman spectra of DNC array and Si wafer are measured at 532 nm excitation wavelength. Results are shown in Fig. 6a. As can be seen the Raman intensity of DNC array is fivefold stronger than that of Si substrate. This enhancement is attributed to the resonantly enhanced optical fields.

Furthermore, the reflectance spectra for a DNC array as well as for bare flat Si surface are measured and shown in Fig. 6b, for comparison. It can be seen that the DNCs array reduces the reflectance of the Si wafer over the entire spectral wavelength range. The diameter of NC cross section decreases from base to the apex thus, the optimum resonance condition can be established for different wavelengths. Therefore, a decrease in reflectance i.e. a high broadband absorbance is observed in the whole spectrum^30^. Reduction in the reflection power is also caused by strong forward coupling of light into the high index substrate by Mie resonances^31,32^. The electric field distribution in Fig. 4 (below of all panels) indicates how light is coupled to the substrate. In fact, the substrate provides a leaky channel for the light confined in the NCs.

### Optical mode characteristics: Multipole decomposition method

**Figure 7:**
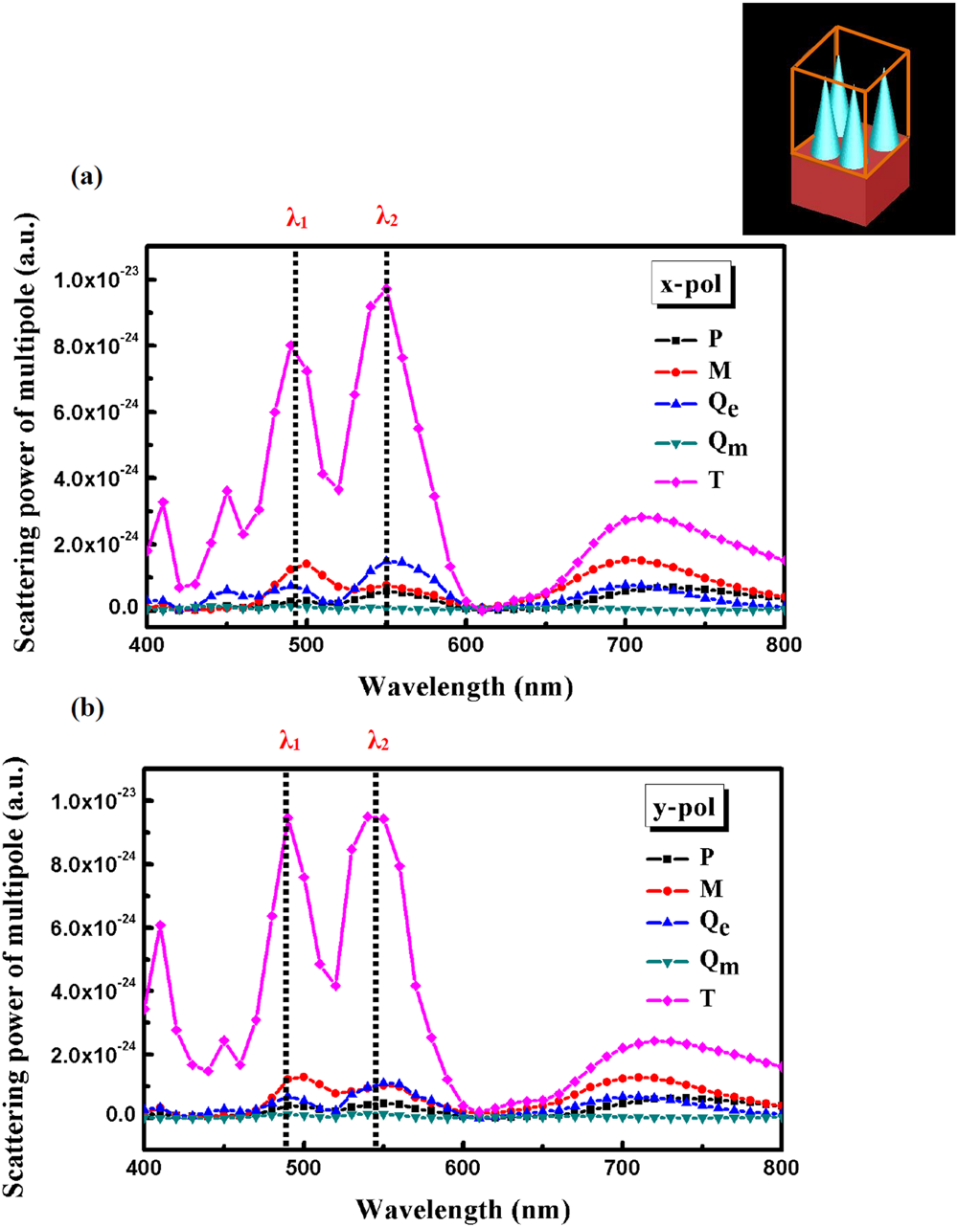
Decomposed scattered power in terms of electric dipolar moment (***P***), magnetic dipolar moment (***M***), electric quadrupole dipolar moment (***Q***_***e***_), magnetic quadrupole dipolar moment (***Q***_***m***_ **)**, and toroidal dipolar mement (***T***) for incident (**a**) X-polarized and (**b**) Y-polarized light. Note that toroidal moment, T, plays a dominant role for all resonances. Calculations are performed in the box demonstrated in the inset, orange color.

The multipole scattering properties of silicon nanostructures have attracted a growing interest in the scientific community, due to their applications in solar cells^33,34^, and in field-enhanced surface spectroscopy^35^. Here, an analytical model for the multipole expansion is used to characterize the nature of each resonant mode in the reflection spectra. In fact, the reflection spectrum is decomposed into radiating contribution of multipoles, including electric dipole (ED), magnetic dipole (MD), toroidal dipole (TD), electric quadrupole (EQ) and magnetic quarupole (MQ). The localized distribution of the volume current density J (in the box demonstrated in the inset of Fig. 7) is used to calculate the multipoles scattering contribution based on the following equations^36–38^:

1 $$electric\,dipole\,moment\,:\,\overrightarrow{P}=\frac{1}{i\omega }{\int }^{}\overrightarrow{J}{d}^{3}r,$$

2 $$magnetic\,dipole\,moment\,:\,\overrightarrow{M}=\frac{1}{2c}{\int }^{}(\overrightarrow{r}\times \overrightarrow{J}){d}^{3}r,$$

3 $$toroidal\,dipole\,moment\,:\,\overrightarrow{T}=\frac{1}{10c}{\int }^{}[(\overrightarrow{r}.\overrightarrow{J})\overrightarrow{r}-2{r}^{2}\overrightarrow{J\,}]{d}^{3}r,$$

4 $$electric\,quadrupoole\,moment\,:\,{Q}_{\alpha \beta }=\frac{1}{i2\omega }{\int }^{}[{r}_{\alpha }{J}_{\beta }+{r}_{\beta }{J}_{\alpha }-\frac{2}{3}(\overrightarrow{r}.\overrightarrow{J}){\delta }_{\alpha \beta }]{d}^{3}r,$$

5 $$magnetic\,quadrupole\,moment\,:\,{M}_{\alpha \beta }=\frac{1}{3c}{\int }^{}[{(\overrightarrow{r}\times \overrightarrow{J})}_{\alpha }{r}_{\beta }+{(\overrightarrow{r}\times \overrightarrow{J})}_{\beta }{r}_{\alpha }]{d}^{3}r,$$

where c is the speed of light, $\overrightarrow{{\boldsymbol{r}}}$ is distance vector from the origin to point (x, y, z), and α, β = x, y, z. Accordingly, the decomposed far field scattering power by the multipole moments can be written as^39^

6 $${I}_{P}=\frac{2{\omega }^{4}{|\overrightarrow{P}|}^{2}}{3{c}^{3}},$$

7 $${I}_{M}=\frac{2{\omega }^{4}{|\overrightarrow{M}|}^{2}}{3{c}^{3}},$$

8 $${I}_{T}=\frac{2{\omega }^{6}{|\overrightarrow{T}|}^{2}}{3{c}^{5}},$$

9 $${I}_{Q}^{e}={\omega }^{6}\,{\sum }^{}\frac{{|{Q}_{\alpha \beta }|}^{2}}{5{c}^{5}},$$

10 $${I}_{Q}^{m}={\omega }^{6}\,{\sum }^{}\frac{{|{M}_{\alpha \beta }|}^{2}}{40{c}^{5}},$$

The radiated powers of multipole moments as a function of wavelength are calculated using equations 1–10, and results are shown in Fig. 7. As seen, all multipole moments contribute in the scattering power at a broad wavelength range from 400 to 800 nm; however the TD moment has the strongest contribution. Two maximum at wavelengths of λ_1_ and λ_2_ are specified in the scattering curves (Fig. 7) which are related to the different multipole resonances. These wavelengths correspond to the wavelengths specified in Fig. 4a. It should be noticed that, ignoring the effect of substrate in the calculations of multipole moments leads to the discrepancy between wavelength positions of λ1 and λ2 specified in Figs 4 and 7. It is observed that the decomposed power for TD moment is stronger than that of ED (25 and 14 times), MD (5.5 and 12.5 times), EQ (10.5 and 6.5 times) and MQ (64 and 119 times) moments at wavelengths of λ1 and λ2, for incident X-polarized light.

**Figure 8:**
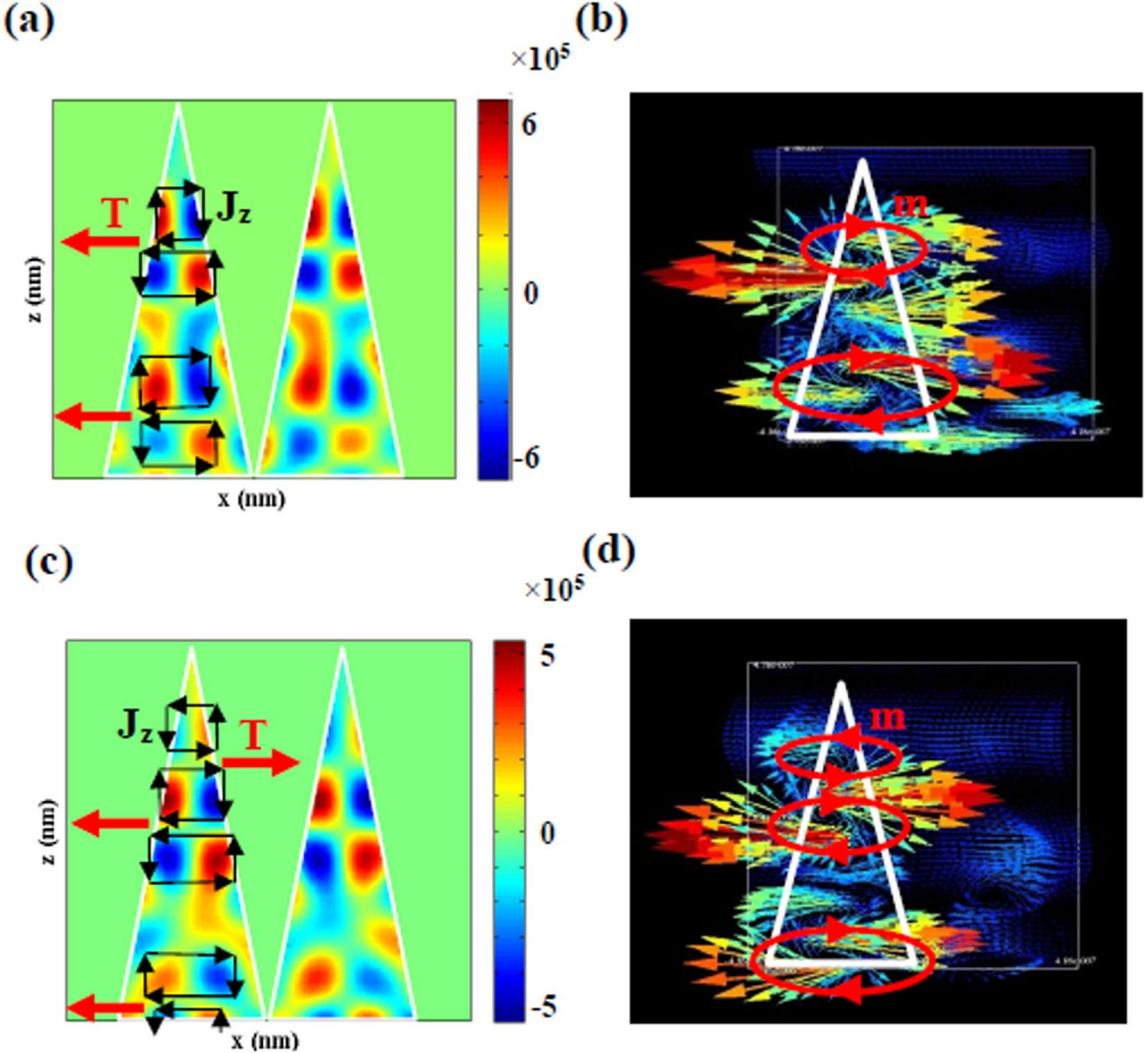
(**a**) Distribution of current density of *z*-component on the xz plane at resonance wavelength of 528 nm for incident X-polarized light. The red and black arrows show the toroidal dipole, T, and current density, J, respectively. (**b**) *H* field vectors, red arrows show the magnetic dipole moments, m, of the silicon NCs. (**c**,**d**) The same as (**a**,**b**) but at resonance wavelength of 578 nm.

To explore the origination of the TD moment, distribution of the current density, j_z_, (at resonance wavelengths λ1 and λ2) is simulated and displayed in Fig. 8a and c, the current density directions are shown with black arrows throughout the cone. It is seen that the displacement currents have opposite directions inside the cones. Actually, opposite direction current densities originate from variation of the cone’s cross sectional area from base to the apex. The magnetic field vectors in the y-z cross section are displayed in Fig. 8b and d. It is seen that the magnetic field vectors have a head to tail distribution in the y-z cross section^40^. On the other hand, ring-like profiles of the magnetic fields are formed and shown with the red arrows in Fig. 8b and d. These ring-like profiles of magnetic dipoles create TD resonances which are shown with the red arrows in Fig. 8a and c ^39^. The significance of the TD moment was established some years ago^41^. It was observed only in the metamaterials^42^ which produces many interesting properties such as nonreciporocal refraction^24^ and magnetoelectric effect^25^. It should be noticed that our Si-DNC array which provide significant TD moment, can be fabricated more easily than the toroidal metamaterial nanostructures reported before^36,43–45^.

### Optical response characteristics of Ag modified DNC arrays

**Figure 9:**
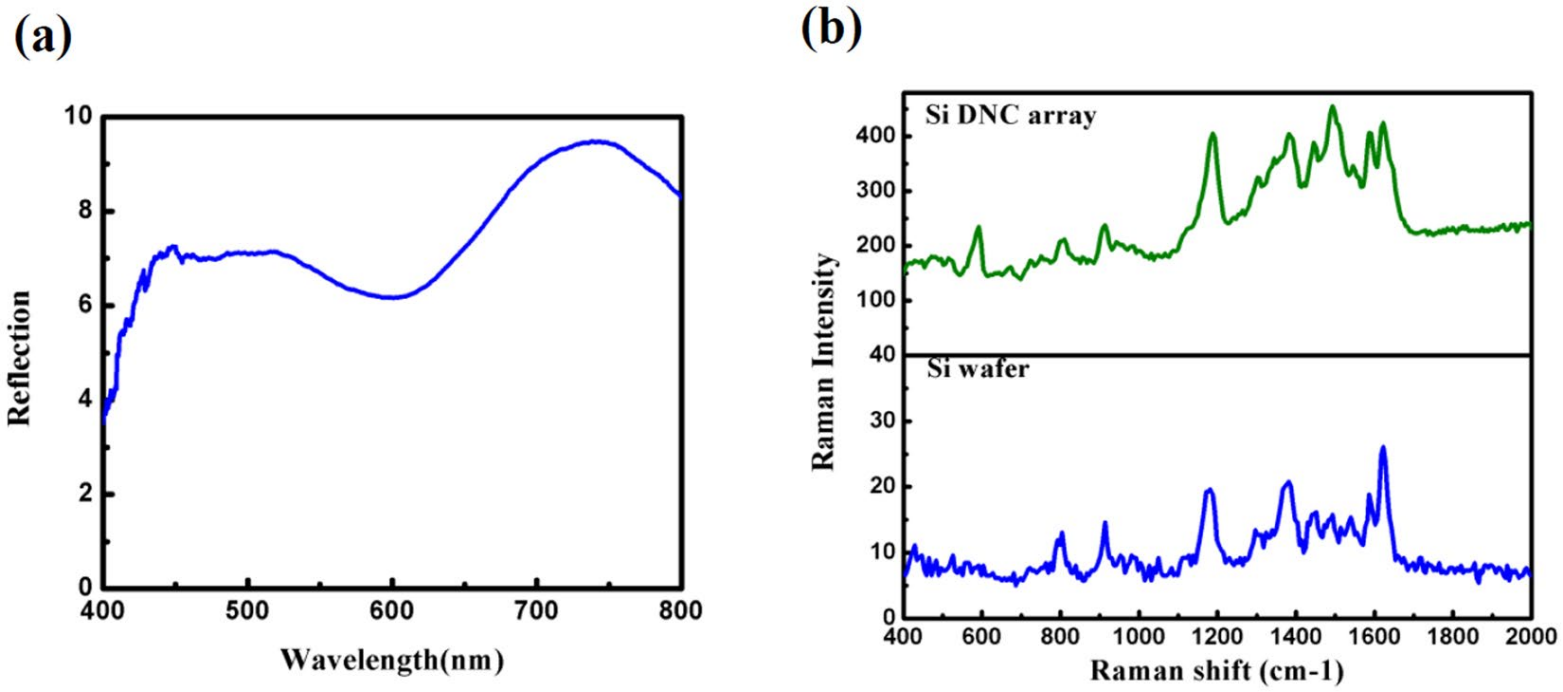
(**a**) The reflection spectrum of DNC array decorated with Ag NPs for deposition time of 20s, (**b**) SERS spectra of two types of substrates including DNC array decorated with Ag NPs (DNC/Ag NPs array) and crystalline silicon wafer decorated with Ag NPs (silicon/Ag NPs) with deposition condition similar to DNC/Ag NPs array.

As already discussed, in this study, the DNC array were deposited using electroless deposition method. This method leads to the formation of a conformal thin film of Ag NPs, for which the size of NPs decreases from tip to the base. The SEM images for such a substrate after deposition are shown in Fig. 3. The influence of the Ag NPs decoration on the optical properties of silicon DNC array is investigated. For this aim, the reflection spectrum of DNCs/Ag NPs substrate is measured and shown in Fig. 9a. It is seen that the reflection dips for the Ag decorated DNC array is much broader than those obtained for the bare substrate, furthermore, they merge together.

**Figure 10:**
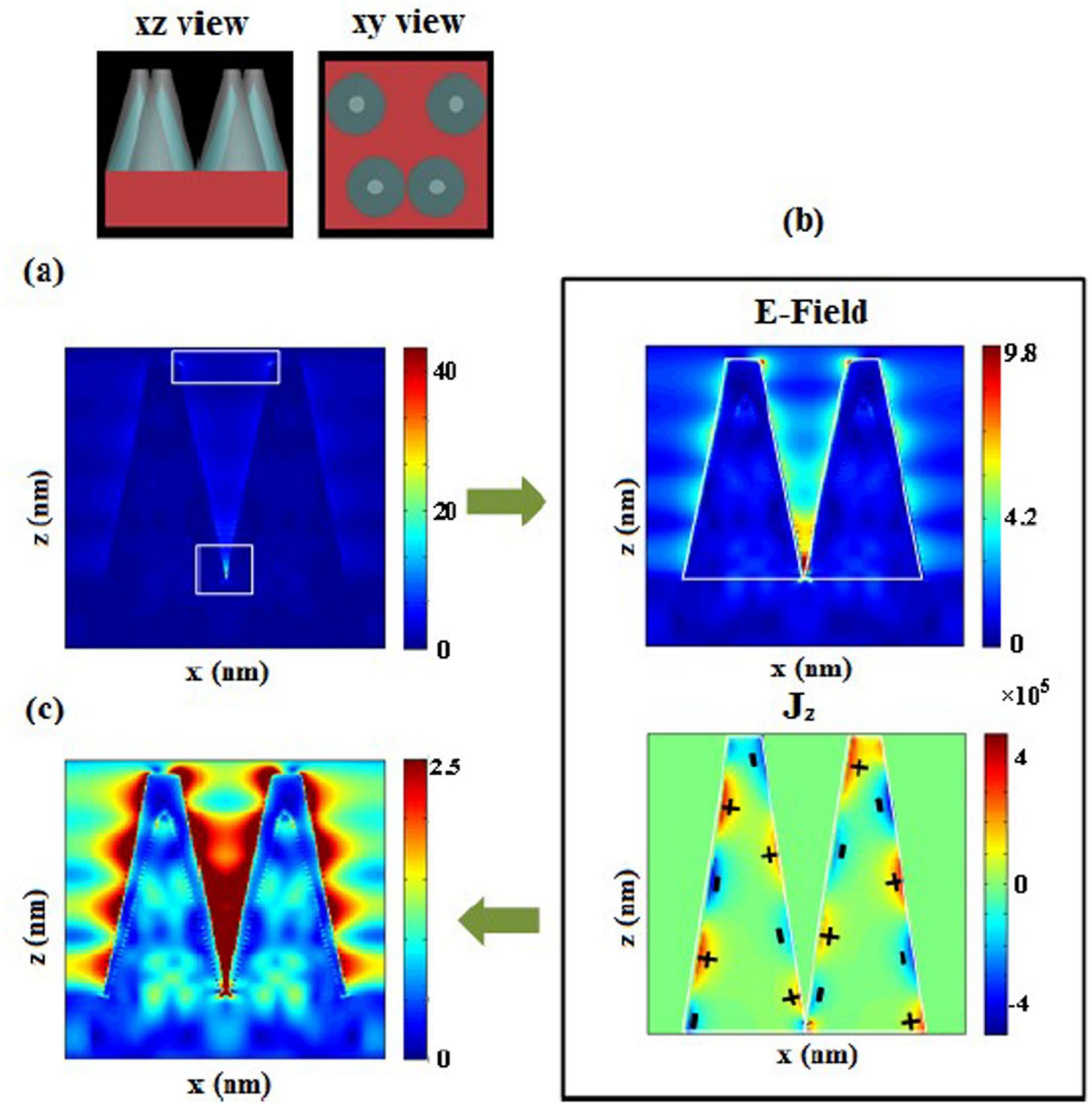
The cross section E- field maps for incident X-polarized light at 532 nm excitation wavelength, for (**a**) electric field range of 0–40, (**b**) 0–9.8, distribution of current density of *z*-component on the xz plane which indicate the existence of LSPR longitudinal standing wave, and (**c**) 0–2.5. It is seen that both electric and magnetic field resonances are simultaneously excited. Below is enlargement of the Si layer.

As we have already mentioned, Ag NPs with larger size lye on the NC tips and smaller size NPs lye on the NC sidewalls. Thus, for simplicity, in the numerical simulation of the optical response, it is assumed that a thin Ag layer is deposited through electroless deposition, whose thickness decreases from NC’s tip (40 nm) to base (1 nm) to apply the effect of changes in the size of NPs. NP with approximately 40 nm in size is measured from the SEM images. To characterize the optical properties of this nanostructure as a SERS substrate, the near electric field distribution at excitation wavelength of 532 nm under incident X- polarized light is simulated and is demonstrated in Fig. 10. To reveal all resonances which may contribute in the enhancement, we have depicted the results of our numerical simulations for a several range of near electric fields. As specified in Fig. 10a, it is seen that the electric field enhancement is more significant in the gap between double NCs and sharp tips of metallic cones. The LSPR longitudinal standing wave is also excited in Ag thin layer on the Si NCs sidewalls as illustrated in Fig. 10b (lower electric field range)^46,47^. In this regard, distribution of current density is calculated and shown in Fig. 10b, which confirms the existence of the longitudinal resonance on the Ag thin layer. Similarly, decreasing the electric field range shows presence of the Mie resonances inside the Si-DNC array with lowest strength.

It is demonstrated that maximum near electric field strength at hot spots is the main factor which determine the amount of Raman signal enhancement factor (EF)^48^. So, distribution of electric field is calculated at the excitation wavelength of 532 nm and demonstrated in Fig. 10. to calculate the EF, the ${|\frac{{E}_{loc}({\omega }_{ex})}{{E}_{in}({\omega }_{ex})}|}^{4}$approximation commonly is used^49,50^, whereas ${E}_{loc}({\omega }_{ex})$ is maximum local electric field at the excitation wavelength. Our calculation indicates that the EF of our Si-DNC array (by considering a thin Ag layer on the NCs) is in the order of 2.5 × 10^6^.

Actually, there are conformal thin film of Ag NPs on the top and sidewalls which play an important role in the SERS measurements. In fact, interplay of multiple resonances including localized surface plasmon resonance (LSPR) of Ag NPs, longitudinal standing wave resonance of Ag layer, LSPR in the tip of nanocones and inter-particle interaction in the gap region give rise to significant local electromagnetic field enhancement. We believe that the presence of Ag NPs in practice is responsible for the discrepancy observed between experimental EF (is given in the next section) and the calculated EF.

### Evaluation of the capability of DNC array as a SERS platform

To evaluate the SERS capability of DNCs array, 15 µL CV solution (1µM), is directly placed on the substrate and allowed to dry. The SERS measurements are performed under 0.5 mW excitation power with integration time of 1s and results are shown in Fig. 9b. It is seen that DNC/Ag NPs array provides strong SERS signal. Actually, each Ag NP plays as an active site which increases the Raman signal. Moreover, interaction between multiple resonances in the gap regions creates hot spots which also lead to the enhancement of Raman scattering. Sharp-tips DNC array are another factor which provides more hot spots per unit surface area for the extraordinary enhanced Raman scattering effect.

**Figure 11:**
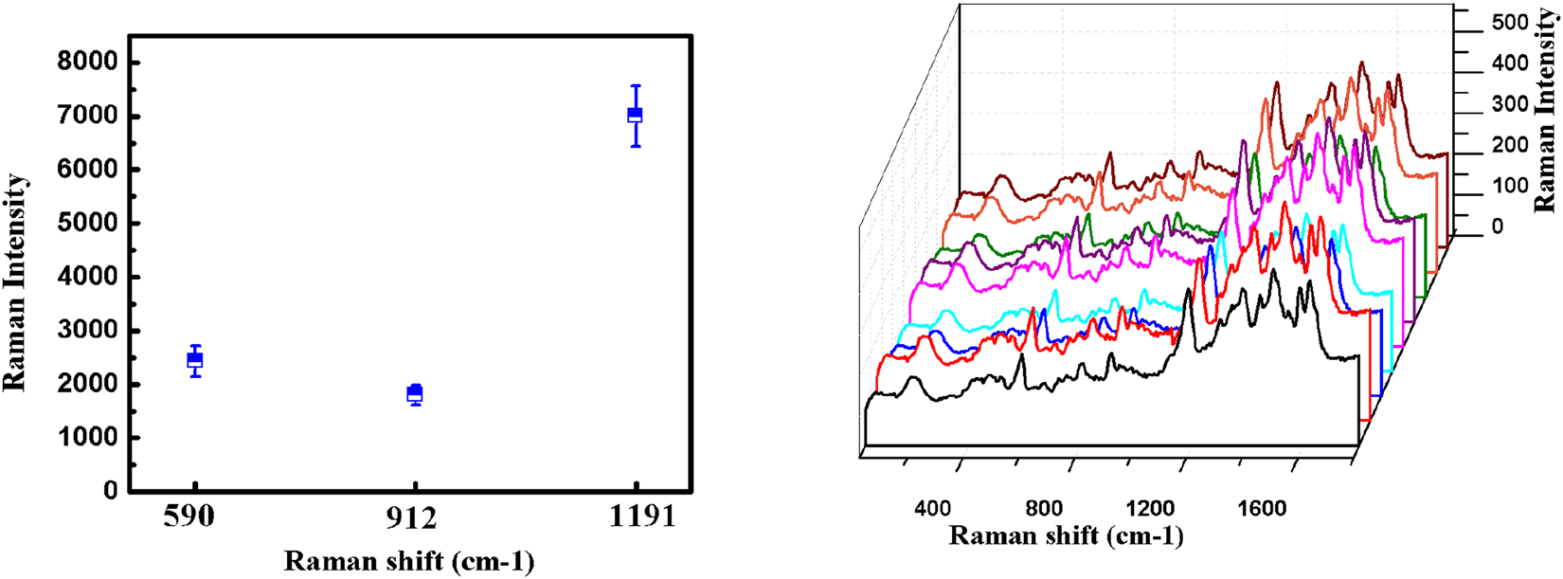
(**a**) The SERS intensity of CV molecules at 590 $c{m}^{-1}$, 912 $c{m}^{-1}$ and 1191 $c{m}^{-1}$ Raman lines with RSD values of 8.87%, 9.32%, and 8.80%, respectively. (**b**) The SERS spectra of CV molecules at nine different points to evaluate reproducibility of substrate.

Reproducibility is crucial for the SERS detection. To investigate the uniformity and reproducibility of our 3D DNC array substrate, multiple measurements are performed under the same experimental conditions.The calculated relative standard deviation (RSD) values for vibrations at 590 cm^−1^, 912 cm^−1^ and 1191 cm^−1^ (Fig. 11) are 12%, 9.9%, and 8%, respectively. Results clearly demonstrate that this Ag modified Si nanostructure is a suitable substrate for highly reproducible SERS measurements. This uniformity and reproducibility can be attributed to the uniformity of the nanostructure including uniformly aligned DNC array and evenly distributed Ag NPs on the sidewalls of NCs.

### Comparison between different substrates

In order to evaluate the capability of our fabricated DNCs/AgNPs array, Raman spectra for three different substrates including DNCs, DNCs/Ag NPs and crystalline silicon/Ag NPdecorated substrates are measured and compared with each other. The crystalline silicon is identically deposited with Ag NPs using the electroless deposition method, for comparison purposes. The SERS spectra for the three substrates are measured for the same volume and concentration of CV solution (15 µL and 1 µM respectively) under 0.5 mW excitation power and integration time of 1s. Figure 9b shows the Raman spectra for two substrates (DNCs/Ag NPs and crystalline silicon/Ag NPs). In the spectrum of bare DNC array substrate (not shown), only fluorescence and Raman line of silicon at 520 $c{m}^{-1}$are seen, and no Raman signal enhancement is observed. Figure 9b shows that the SERS signal for the crystalline silicon substrate is 22 times weaker than the SERS signal for the DNCs substrate (obtained from CV Raman line at 1191 $c{m}^{-1}$). This enhancement is related to the larger number of active sites in the confocal volume in comparison with the flat substrate. So, more EF is achieved from DNCs/Ag NPs, making it an appropriate SERS substrate.

### Sensitivity estimation

**Figure 12:**
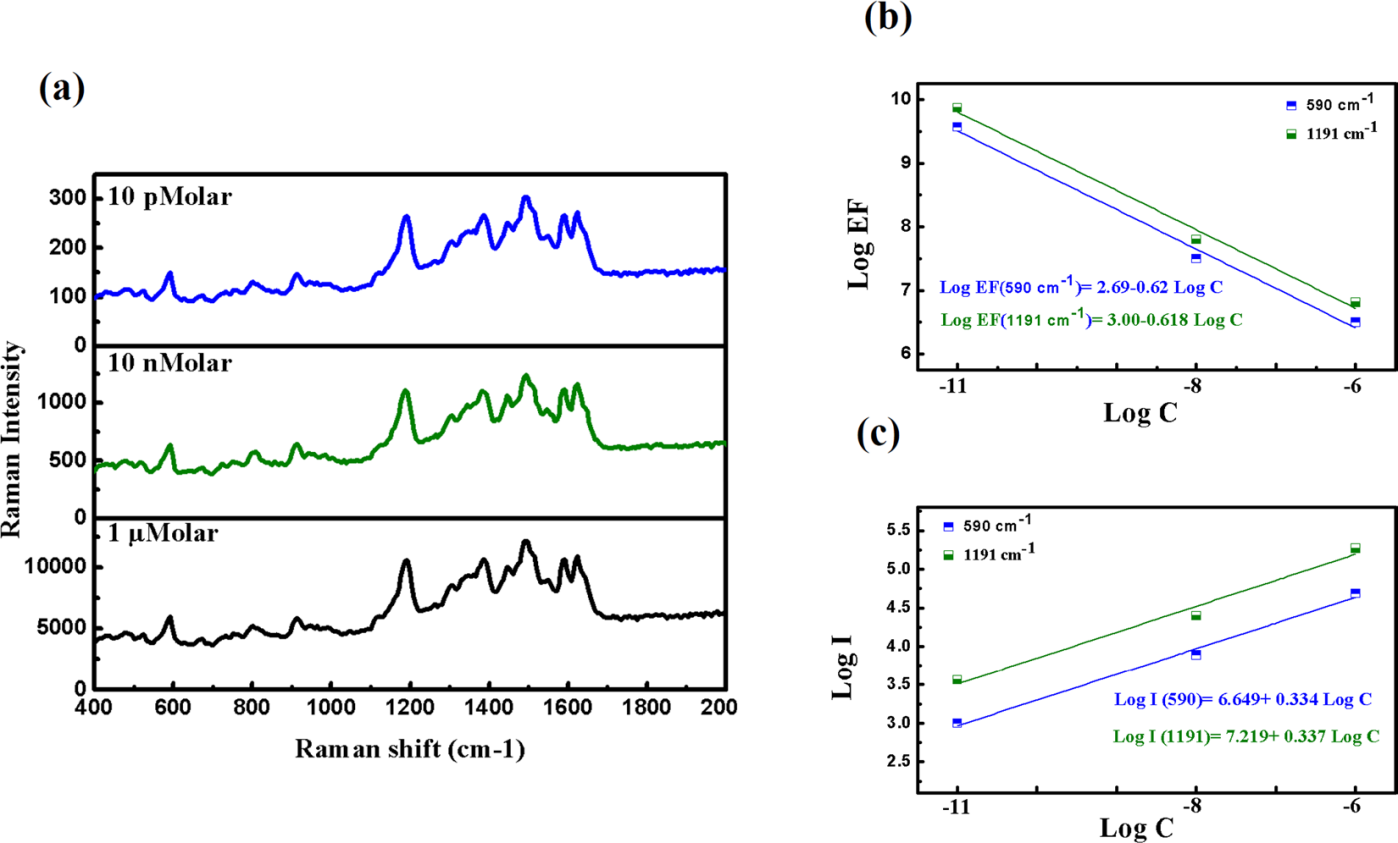
The SERS spectra of CV molecules for the concentration of ${10}^{-6}$, ${10}^{-8}$ and ${10}^{-11}$ M on the DNCs/Ag NPs array SERS substrates with power 2 mW and integration time of 10 s. (**c**) log-log plot of Raman intensity peak at 590 $c{m}^{-1}$ and 1191 $c{m}^{-1}$ versus CV concentration. (**c**) log-log plot of Raman scattering EF for peak at 590 $c{m}^{-1}$ and 1191 $c{m}^{-1}$ versus CV concentration.

To estimate the sensitivity of DNC/Ag NPs array substrate, the SERS spectra for different concentrations of CV solution are measured employing an excitation power of 2 mW and integration time of 10s. For this aim, 15µL of the CV solution with various concentrations of 10 pM, 10 nM and 1 uM, is dropped on the substrate. Then, the SERS measurements are performed. Results are shown in Fig. 12. We precisely calculate the EF by comparing the intensity of two CV Raman peak at wavenumbers of 590 cm^−1^ as well as the 1191 cm^−1^ with the reference. For this calculation, first the fluorescence baseline is removed using iterative multi polynomial fitting algorithm^51^. The experimental EF value is calculated using^52,53^:

11 $$EF=\frac{\frac{{I}_{SERS}}{{N}_{surf}}}{\frac{{I}_{ref}}{{N}_{bulk}}},$$

where, I_SERS_ is the SERS intensity of the selected peak, N_surf_ is the number of molecules contributes to the measured SERS signal, I_ref_ is the Raman intensity of the selected peak from the reference spectrum, and N_bulk_ is the number of molecules contributing to the measured reference spectrum. The number of molecules is calculated by:

12 $$N=\frac{{N}_{A}CV{A}_{laser}}{{A}_{spot}},$$

where, C is the molar concentration of the CV solution, ${A}_{spot}$ is the total area of the CV molecules on the substrate, ${A}_{laser}$ is the area of the excitation laser spot, V is the volume of the CV drop and ${N}_{A}$is the Avogadro’s number.

As seen, the Raman bands for the CV molecules can still be identified from the concentration down to 10^−11^ M. The calculated EFs of the DNC array for different concentrations of CV solutions are shown in Fig. 12b and the results are summarized in Table 1. It is seen that the EF value of 7.47 × 10^9^ is obtained for concentration of 10^−11^ M. This high EF value indicates that the AgNPs/DNCs array is a suitable SERS substrate with very high sensitivity. Our results indicate that the integrated intensity and EF value increases non-linearly with its molar concentration as explained in our previous work.

**Table 1:** Enhancement Factors calculated from different concentrations.

concentration	1 µM	10 nM	10 pM
EF_590_	3.17 × ${10}^{6}$	3.1 × ${10}^{7}$	3.73×${10}^{9}$
EF_1191_	6.81 × ${10}^{6}$	6.2 × ${10}^{7}$	7.47 × ${10}^{9}$

## Conclusion

In summary, we described fabrication of the Si-DNC array, and explained its applicability as a SERS substrate when it is decorated with Ag NPs. First, the optical response of the bare Si-DNC array is studied both theoretically and experimentally. Our simulation results indicate that the TD moment can be excited in the Si NCs over a broad wavelength range of optical frequencies, using the multipole scattering theory. It is shown that, variation of cone diameter from the cone’s tip to the base, lead to formation of current densities with opposite directions and consequently H-field vortexes inside the nanocones. After that, the SERS characteristics for substrate are studied. It is demonstrated that the DNC/Ag NPs array provides high density of hot spots which leads to the significant SERS enhancement, so it may serve as a suitable substrate for practical applications such as biosensing and biodetection. We have shown that the SERS enhancement results from combination of multiple resonances. Finally, EF in the order of 7.47 × ${10}^{9}$ and limit of detection of 10^−11^ M are obtained.

## Methods

### SERS measurements

To interrogate the SERS property of the DNCs/Ag NPs substrate, crystal violet (CV) is used as a target analyte. First, 15 µL CV is dropped on the substrate and let it dry. Then SERS measurements are made using a microscope Raman spectrometer (Teksan_N1-541, Iran) with the resolution of 1 cm^−1^ and excitation wavelength of 532 nm.

### FDTD Simulation method

Finite-difference time domain (FDTD) method is performed to simulate the reflectance spectrum and near electric and magnetic field distributions of nanostructure. Periodic boundary condition in the x and y directions, and perfectly matched layers (PML) in the z direction are used to eliminate spurious reflections. In all cases, optical constants of materials are taken from Palik handbook^54^. The geometrical parameters are taken from SEM images. The bottom diameter of the DNC is 290 nm, the height is 780 nm, and the gap is 23 nm. Analogy between simulated and experimental reflection spectra is used to verify the validity of the model.
